# Clinics

**Published:** 1874-10

**Authors:** 


### Clinics.

***Rush Medical College.*** Clinic for Neuropathology, by Dr. WALTER HAY. Reported by HENRY W. HARRINGTON.

CASE OF IDIOCY, RESULT OF EPILEPSY.—Girl, aged eight years. The mother states that the child was apparently well when born, and up to the age of eighteen months was well and bright. She talked, but had never walked. At the age of eighteen months, she began to have epileptic attacks, and they have continued ever since, sometimes recurring several times daily. No hereditary history of epilepsy or other nervous disorder. Soon she seemed to lose the faculty of talking, and has not talked any since. Has been and is perfectly helpless, requiring constant care. On examination, find the patient poorly nourished, skin pale and anæmic; pupils dilated; conjunctivæ pearly; tongue pale, but clean; appetite good; bowels regular; limbs small and poorly developed, but symmetrical; there is no paralysis, the patient being able to move the hands and feet readily; in fact, she is in constant motion, throwing her limbs around, and constantly striking her hands against her teeth, the mouth being open and saliva dribbling away; and articulates indistinct, meaningless sounds. Has a vacant, staring expression of the face—head of good size and well formed.

*Remarks.* We have here a case of idiocy and lack of development of the body, due to arrest of development of the brain and spinal cord, this arrest being caused by the long continued and frequently repeated attacks of epilepsy. There are no signs, nor is there any history of organic infantile paralysis. I think the case is hopeful; would advise the mother to send the child to the Jacksonville Asylum, where she can have special care and training. Ordered chloral hydrate to quiet the child and procure sleep; also nourishing diet; fresh air, and to be placed in the company of other children as much as possible.

CASE OF INCIPIENT CHOREA, WITH TENDENCY TO INSANITY.—Boy, aged seventeen years; student. States that he was well until eighteen months ago; has practiced masturbation for several years; has not had a sufficient quantity of good food, and took very little exercise; commenced feeling weak and tired; had a dull pain in the back and sides; lost his appetite; bowels became costive; had frequent desire to urinate, sometimes passing his water twenty-five times daily; has suffered for some months with involuntary irregular twitchings of the muscles.

On examination, find the patient pale and anæmic; tongue pale and tremulous; sight good; hearing at times defective; pulse, seventy and feeble; temperature, $98\frac{3}{5}°$; respiration, twenty; patient seems dull, and indisposed to mental or bodily exertion; extremities are cold; there is a history of insanity in his family.

*Remarks.* In this case we have an inherent tendency to disorder of the nervous system, only awaiting some exciting cause to assert itself. The patient's habits of over study and deprivation of proper exercise and food, combined with the abnormal irritation, caused by the habit of masturbation, have served to impoverish the nervous centres, this being first manifested by instability of motor nerve cells, and consequent irregularity in muscular action, the same in kind but not in degree, as occurs in fully developed chorea. The intellect is also impaired; he is subject to morbid sensitiveness; exaggerates his troubles and is easily discouraged; there is an innate instability of the nervous system, and a tendency to insanity and convulsive forms of disease. The treatment in this case must be largely moral. He must stop the bad habit. If necessary to bring this about, blister the penis. He should have nutritious diet; tonics, as iron and strychnia; also active occupation in the open air, in order to develop the bodily strength and vigor.

Case of Chronic Myelitis.—Man, aged forty years; railroad laborer. States that he has been much exposed to cold and wet, and has been obliged to work in a stooping position; was well until one and a half years ago; commenced having pain in the back, increased by motion, also shooting pains in the legs; says his legs felt stiff and numb, and his feet heavy. The pain in the back was not severe when he was lying down; he has also been troubled by involuntary twitchings of the muscles of the legs; says that it seems as if the sight in the outer vertical half of the left eye was destroyed. Appetite fair; bowels sometimes loose, at others costive; he has continued getting worse up to the present time.

On examination, patient well nourished; tongue normal; pupils symmetrical; pulse, eighty; temperature, 98⅗° ; respiration, eighteen; patient has trouble in walking, drags his toes; reflex excitability is much increased ; there is tenderness on pressure of the spine over the upper three or four lumbar vertebræ ; intellection is not impaired.

*Remarks.* This, gentlemen, is a somewhat complicated and obscure case. The patient, after exposure to cold and wet, and undergoing severe muscular labor, requiring continual stooping, is attacked by pain in the back, shooting pains in the legs, and loss of sensation—also difficulty in walking, his toes dropping, and catching easily, showing that the muscles are partially paralyzed, the extensors more than the flexors. Evidently the anterior columns of the cord are diseased, but in what form? It is probably a case of inflammation of the spinal cord, gradually extending from before, backwards, involving the posterior columns to some extent, as is indicated by the perverted sensations, and increased reflex excitability. This inflammation has resulted in either induration or softening of the substance of the cord, it is impossible to say which. There is also some degree of meningitis indicated by the pain in the back being increased by motion, and by the involuntary jerkings of the muscles. The main indication to be fulfilled in treatment, is to diminish the amount of blood sent to the cord. For this purpose we order—R. Fld. ext. secale cornut. dr. j, once daily at first, to be increased if necessary. This operates by stimulating the vaso-motor nerves, and exciting contraction of the muscular coat of the blood vessels, and, consequently, diminishing their calibre and the amount of blood sent to the parts, its action being chiefly directed to the spinal cord.

